# 3D bioprinting: novel approaches for engineering complex human tissue equivalents and drug testing

**DOI:** 10.1042/EBC20200153

**Published:** 2021-08-06

**Authors:** Judith Hagenbuchner, Daniel Nothdurfter, Michael J. Ausserlechner

**Affiliations:** 1 Department of Pediatrics II, Medical University of Innsbruck, Innsbruck, Austria; 2 Department of Pediatrics I, Medical University of Innsbruck, Innsbruck, Austria

**Keywords:** bioink, extrusion printing, microjet, replacement of animal experiments, tissue engineering, tissue on chip

## Abstract

Conventional approaches in drug development involve testing on 2D-cultured mammalian cells, followed by experiments in rodents. Although this is the common strategy, it has significant drawbacks: in 2D cell culture with human cells, the cultivation at normoxic conditions on a plastic or glass surface is an artificial situation that significantly changes energy metabolism, shape and intracellular signaling, which in turn directly affects drug response. On the other hand, rodents as the most frequently used animal models have evolutionarily separated from primates about 100 million years ago, with significant differences in physiology, which frequently leads to results not reproducible in humans. As an alternative, spheroid technology and micro-organoids have evolved in the last decade to provide 3D context for cells similar to native tissue. However, organoids used for drug testing are usually just in the 50–100 micrometers range and thereby too small to mimic micro-environmental tissue conditions such as limited nutrient and oxygen availability. An attractive alternative offers 3D bioprinting as this allows fabrication of human tissue equivalents from scratch with hollow structures for perfusion and strict spatiotemporal control over the deposition of cells and extracellular matrix proteins. Thereby, tissue surrogates with defined geometry are fabricated that offer unique opportunities in exploring cellular cross-talk, mechanobiology and morphogenesis. These tissue-equivalents are also very attractive tools in drug testing, as bioprinting enables standardized production, parallelization, and application-tailored design of human tissue, of human disease models and patient-specific tissue avatars. This review, therefore, summarizes recent advances in 3D bioprinting technology and its application for drug screening.

## Introduction

The development of different 3D printing technologies and their adaptation for biological materials have opened a completely new field for tissue engineering that has led to the development of organ-like tissue equivalents and heads forward to print whole organs in near future. This evolution is driven by two major needs: the lack of donor organ for transplantation and the demand for precisely assembled tissue equivalents to study on one hand complex intercellular processes during, e.g. organ morphogenesis and on the other the effects (and side effects) of drugs on human tissue. Human cells encapsulated in 3D spheroids made of Matrigel, collagen or other bio-matrices are increasingly used as 3D tissue models, and this technology is also the basis for growing organoids as avatars of human organs. Organoids provide significant insights in cell sorting, tissue development and morphogenesis, but are limited in size and completely rely on cellular self-organization. This means that morphogenesis cannot be steered, which leads to non-reproducible structural features.

To define the morphology and substructure of designed tissues, additive manufacturing techniques such as filament deposition modeling (FDM) or stereolithogaphy (SLA) printing were adapted to create tissue equivalents that reflect the anatomy and mimic the biology of human tissue. Based on a computer-aided design (CAD) model or medical imaging data, cells, extracellular matrix proteins, cytokines, growth factors or drugs can be assembled in 3D to produce a bio-inspired tissue structure. Compared with traditional tissue engineering approaches, the advantage of 3D bioprinting therefore lies in the strict spatiotemporal control over the deposition of specific cell types and/or proteins and its reproducibility for medium-throughput tissue manufacturing. In principle, a bioprinting project may be divided into three major aspects:

The bioinks are cell-containing hydrogels consisting of extracellular matrix molecules and components that polymerize upon a certain stimulus, which stabilizes the 3D-tissue structure after printing. The bioink must be optimized for each specific cell type and has to serve a number of different functions such as to provide epitopes for adhesion and migration of cells, a certain stiffness and water content to allow diffusion of oxygen and nutrients for cell survival and to maintain the architecture of the bioprinted tissue equivalent. Bioinks can be also combined with cell-free inks consisting of (bio)materials that either serve structural functions (provide e.g. tensile strength) or are sacrificial (i.e. they stabilize the construct until it is fully polymerized).

**Figure 1:**
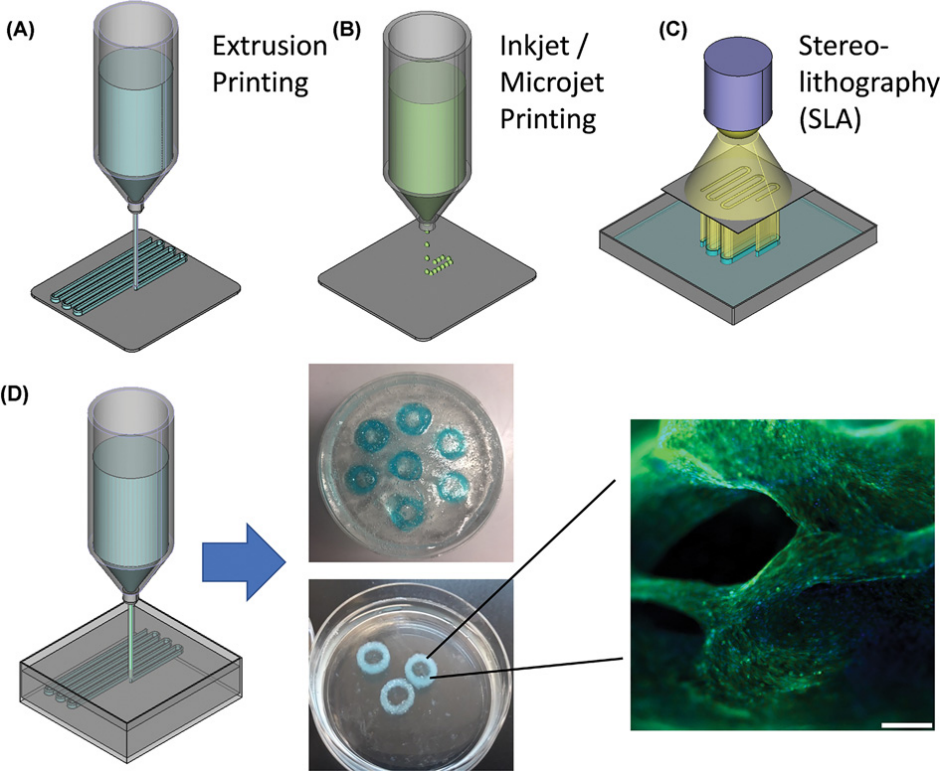
Extrusion, microjet and SLA printing The most frequently used methods for 3D additive manufacturing of living tissue equivalents are (**A**) extrusion-printing, (**B**) inkjet- and microjet- printing and (**C**) stereolithography (detailed description see text). (**D**) A special form of extrusion printing is the ‘freeform bioprinting’, where bioink is injected into a support bath that causes gelation and supports the 3D structure during polymerization. Shown are C2C12 myoblast cells printed in an alginate/GelMA-based bioink using the FRESH method before (top) and after (bottom) liquifying the support bath. After one week of cultivation, C2C12 myoblast cells show 99% viability and are aligned in strands. Green: Calcein-AM vital staining, Blue: Hoechst33342 DNA-stain, Red: propidium iodide staining for dead cells.

In biological labs, the most common bioprinting systems are extrusion- and inkjet/microjet printing systems (or combinations of both). Similar to a plastic filament in FDM-printing the bioink is extruded from the syringe via pneumatic- or piston-driven pressure. For inkjet/microjet printing, the bioink is ejected from the printhead in small droplets, which limits the bioink’s viscosity, but provides a high-resolution, cell-friendly way of depositing single cells, particles and cell conglomerates. The advantage of stereolithography, where light is projected in distinct patterns to polymerize bioink or resin layer-by-layer, is its high resolution. A challenging aspect of stereolithography is the repeated exposure of cells to high-intensity UV light, which hampers cell viability. Further, the sedimentation of cells and particles during the printing process is difficult to control (Figure 1). The pros and cons of different bioprinting technologies are discussed in recent review articles in more detail [1,2].

**Figure 2:**
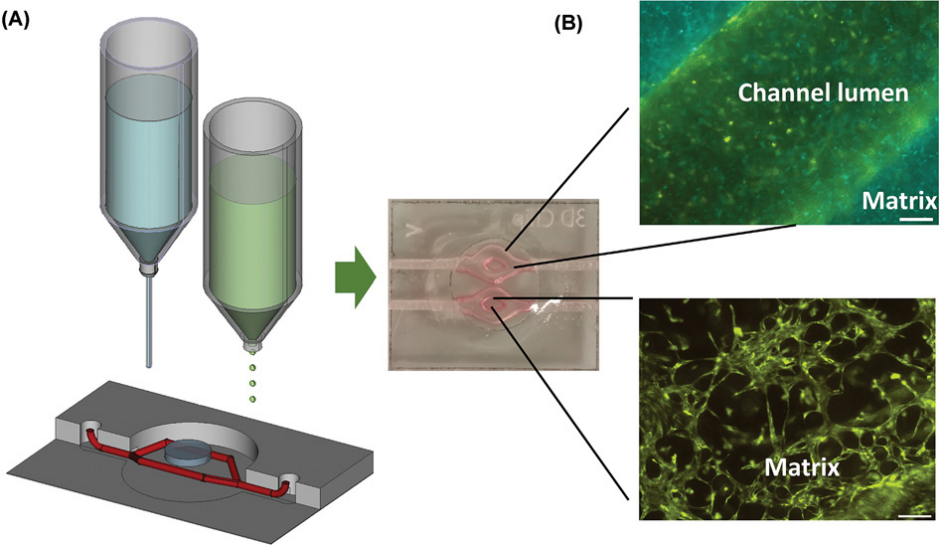
Bioprinting-on-chip for perfused, micro-vascularized tissue-on-chip models By combining extrusion- and microjet-printing techniques, tissue equivalents are printed directly into laser-manufactured acryl/glass chips. Tissues that contain endothelial cell-coated channels were printed using Pluronic F127 as sacrificial support (top). An optimized bioink ell composition induces the spontaneous formation of micro-vessel structures within the hydrogel. Green: EYFP-expressing immortalized human umbilical vein cells, Blue: ECFP-expressing immortalized human fibroblasts.

The third critical point is to keep the printed tissue alive and to mature it into a bio-inspired model that resembles human tissue. Since the living tissue construct will undergo significant changes and self-organization in the *post*-processing phase, bioprinting is a form of ‘4D printing’, where the 3D-fabricated construct changes over time. This means the different cell types present in the tissue model will cooperate and self-organize, migrate, differentiate and produce their own extracellular matrix components. To induce tissue differentiation, the fabricated model has to be exposed to environmental stimuli (e.g. ‘air-lift’ of bioprinted skin) or ‘trained’ to achieve a certain cellular organization (e.g. mechanical /electrical stimulation for muscle constructs). At this point, ‘bioprinting meets mechanobiology’ and provides a plethora of possibilities to investigate, how mechanical stimulation affects intracellular signaling and cellular behavior. To maintain cell viability and function in tissue equivalents beyond diffusion limits (which is around 100–200 µm for oxygen in hydrogels), the transport of nutrients and oxygen to and the removal of waste from each cell has to be guaranteed. Strategies to mimic vasculature via printing conduits for tissue perfusion and the development of bioinks that promote micro-vessel formation during post-processing (Figure 2) are essential to provide efficient molecule exchange throughout the construct and long-term survival of the tissue equivalent.

## Structural information for the 3D tissue model

The 3D design of tissue equivalents will mostly be developed in CAD programs that are provided by the bioprinter manufacturer or are open source such as Blender or FreeCAD. Alternatively, clinical imaging data may be converted into a printable file format. At least from our own experience, there is a continuous cycle of ‘trial and error’ with permanent refinement and adaptation of design, bioink formulation and bioprinting parameters. Structural clinical/biological data (e.g. vessel networks) may be obtained either from specific papers [3–5] or from platforms such as the NIH 3D print exchange (https://3dprint.nih.gov/) which offers models for cardiovascular and neuronal networks. For printing, the CAD file has to be converted into an STL (Surface Tesselation Language / STereoLithography) file. Similar to consumer FDM-printers, the STL-file is then further processed by the printer-specific ‘slicer’ that converts the 3D model into ‘a pile of layers’, the G-code. The G-code defines *xyz* axis movement of the printer-head and the deposition of material at predefined positions by the extruders.

## Bioinks as the crucial components for cell survival, differentiation and structural integrity

Bioinks are defined as cell-containing, bio-compatible hydrogels to print cells and hydrogel matrix in one step. ‘Biomaterial inks’ on the other hand are synthetic or biological materials that are 3D fabricated (without cells) [6]. Examples for biomaterial inks are thermoplasts such as polylactic acid or polycaprolactone, ceramics or bioactive glasses or inks consisting of collagen or chitosan that are printed in acidic pH, which would harm embedded cells. The bioink hydrogel acts as a 3D-scaffold that provides cell adhesion motives and structural integrity. Of note, cells should be also able to degrade this scaffold during *post*-processing as they will have to replace it by their own extracellular matrix (ECM) proteins. The second important property of the bioink is its spontaneous chemical or physical stabilization immediately after being deposited to preserve the desired 3D structure. This might be achieved by a temperature shift (e.g. gelatine or agarose gelling on cold surfaces) by light-induced crosslinking via photoinitiators in combination with methacrylated gelatin or hyaluronic acid or by ionic gelling.

Several points have to be taken into account when developing bioinks for a specific tissue equivalent: Most importantly, the bio-matrix has to provide appropriate motives for the adhesion, survival and migration for the cell types present in the tissue equivalent. In the body, cells receive a plethora of survival signals via growth factors and the adhesion to the matrix is crucial for correctly organizing the cytoskeleton and the proper functioning of the cell. If cells lose contact to the substrate this immediately causes cytoskeleton perturbations and triggers a strong death signal, so called Anoikis [7]. In the body of multicellular organisms, this mechanism of cell death induction is essential for preventing metastasis and maintaining tissue homeostasis – during bioprinting, it represents a significant challenge for the viability of the printed cells. The death signal resulting from matrix detachment can be delayed for some time by pro-survival signaling, but will eventually lead to apoptotic cell death, if the cells do not adhere within very short time to the hydrogel matrix. Different families of adhesion molecules mediate substrate recognition and attachment. Members of the cadherin transmembrane protein superfamily directly connect to actin and intermediate filaments in the cytoplasm and are extracellularly stabilized by Ca^2+^ ions. These proteins interact with each other and extracellular matrix proteins and mediate cell clustering during development [8]. The presence of Ca^2+^ ions in the hydrogel is therefore essential for cellular self-organization via cadherins. The integrin receptor family controls cell–cell and cell–matrix interaction, binds to collagen (and the protease-digested derivative gelatin) and especially to a motive formed by the amino acids arginine, glycine and aspartate (RGD)-motive that is present in the extracellular matrix proteins fibronectin, vitronectin and laminin [8]. Therefore, either natural, protein ECM components that contain binding sites for membrane receptors must be included in the bioink, or synthetic/polysaccharide-based matrix components have to be functionalized with peptides such as RGD to allow adhesion, migration and to preserve viability. Instead of adhesion molecules, matrix components can also be functionalized with protein-mimetic peptides that for example induce angiogenesis or trigger cell differentiation into a specific lineage [9]. Since the composition of ECM-proteins, ECM-bound growth factors and glycosylation patterns significantly influence cell differentiation, another strategy is to generate tissue-specific bioinks from decellularized organs [10,11]. This strategy provides the cells with known (and unknown) ECM-components including bound signaling molecules characteristic for the tissue of interest. Although such decellularized ECM-based bioinks may be the best choice for a bioprinted tissue equivalent, to dissolve the tissue-derived ECM it has to be proteolytically digested, which in turn will destroy many ECM-anchored signaling molecules. In one recent paper, this problem was circumvented by milling and filtering ECM to µm-sized particles [12], which can be printed and are expected to represent more ‘native’ ECM than e.g. pepsin-digested decellularized matrix.

Ideally, the bioink and resulting tissue equivalent should have mechanical properties similar to the native human tissue and these rheo/mechanical properties are significantly influenced also by the polymerizing component of the bioink. The major challenge with at least extrusion- and inkjet-printing-based methods is that the bioink should be of low viscosity as long as cells and bio-matrix are in the syringe, but has to polymerize immediately after leaving the nozzle tip to stabilize the printed construct. A number of different strategies to polymerize bioinks were described – the most frequently used ones are chemical crosslinking by bivalent ions or enzymes, light-induced polymerization or temperature/pH-change-induced stabilization. Examples for chemical crosslinking are alginate, a polysaccharide from algae that spontaneously polymerizes in presence of bivalent ions such as Ca^2+^ (Figure 1) or fibrinogen, which is cleaved by thrombin and polymerizes to fibrin-networks [13,14]. The most commonly used bioink component, however, is chemically modified gelatin, so called gelatin-methacrylate (GelMA). Gelatin as a product of proteolytic collagen degradation has much weaker mechanical properties than collagen, but is in contrast well soluble at 37°C and still contains adhesion motives for integrins, cleavage sites for metalloproteinases and can be covalently crosslinked by UV-activated photoinitiators [15]. Besides gelatin, many other natural and synthetic bioink components such as collagen, hyaluronic acid or polyethylenglycol can be methacrylated and thereby covalently polymerized [4,16]. Temperature-induced gelling as in the case of low-melting agarose or gelatin were the earliest strategies to generate 3D structures, but are critical in every-day-lab-use as environmental conditions have to be strictly controlled. Nevertheless, there are a number of successful studies using these methods – an impressive example is the printing of functional bioprosthetic ovaries [17]. Collagen and chitin/chitosan are soluble only at low pH as hydrogen-bonds between the protein-chains are protonized leading to dissociation. Rapid change of pH causes spontaneous reassembly of native conformation and gelling, which can be exploited for printing of complex collagen structures like heart valves [5].

## Bioprinting methods and procedures to build complex tissue equivalents for tissue regeneration and disease modelling

The extrusion and microjet printing methods are the most widely available bioprinting platforms and the most versatile ones. As already outlined above, similar to FDM-printing, the extrusion printing process dispenses bioink ‘filament’ through a needle via pneumatic or piston-driven pressure. Overhanging or hollow structures, however, will immediately collapse during additive printing, which can be only prevented when using a sacrificial support ink such as Pluronic F127. After crosslinking, the construct is cooled down to approximately 15°C, which liquefies Pluronic so that it can be removed from the hydrogel. With this widely used method for printing channels and chambers in hydrogels, among many other complex tissue equivalents a three-layered skin equivalent consisting of epidermis, dermis and hypodermis for perfusion was generated [18]. Alternatively, as demonstrated by Merceron et. al. it is also possible to combine biodegradable thermoplasts (e.g. polycaprolactone (PCL) or polyurethane (PU)) with cell laden hydrogels to generate living tissue structures such as a muscle–tendon interface, where the thermoplastic material provided the stability for the tendon surrogate [19]. Of note, the combination of thermoplastic material such as PCL with cell-laden bioink faces significant challenges related to the hot extrusion of the PCL at 60–70°C which, wherever the extruded filament touches cell-containing hydrogel during printing will significantly hamper cell viability. For possible tissue regeneration approaches, the implantation of such composite materials and their slow degradation in the body might cause immune responses, encapsulation and interfere with tissue regeneration. Instead of printing support structures and bioink sequentially, co-axial extrusion needles were developed to print strings of two different bioink formulations in ‘core–shell’ structures. With this technology, introduced by the Ozbolat group, it is possible to generate hollow tubes by extruding e.g. the gelling solution via the inner (core) needle and bioink through the (outer) shell needle [20]. The team used this method to manufacture complex tubular networks, where the inner wall is formed by endothelial cells and the outer shell consists of smooth muscle cells [21].

An alternative to the layer-by-layer manufacturing is the so-called “freeform bioprinting” that allows the construction of tissue equivalents without support material [22]. Layer-by-layer bioprinting is somehow limited in the achievable complexity and it is not possible to directly assemble cells or mini-spheroids in 3D. This problem can be solved by injecting the bioink structures into a ‘support bath’ that on one hand causes the gelation of the bioink and on the other supports the 3D structure until full polymerization. The support material needs to serve three functions: First, it is solid-like under static conditions and holds the printed structure in place, but fluid-like when shear stress is applied by a needle moving through the bath, second, it induces spontaneous gelling of the bioink and third, it can be removed at physiological parameters that do not harm cells. Support matrices made of gelatin-microparticles that melt at 37°C (Figure 1) [5,23], or of alginate/xanthan gum that is enzymatically degraded [24,25] have been described. With these methods it was possible to 3D bioprint functional heart valves [5], a full-size heart model [3] and even a living ‘mini-heart’ with natural architecture, where the cardiac parenchym consisted of induced pluripotent stem cell (iPSC)-derived cardiomyocytes and major blood vessels were coated with iPSC-derived endothelial cells [25]. This demonstrates the potential of freeform bioprinting approaches to manufacture patient-specific tissues and functional organs in future. A major challenge when printing large volume tissue with this technology is cellular access to sufficient nutrients and oxygen. Choi et al. demonstrated an elegant solution for large muscle constructs: they found that the inner regions of muscle constructs became necrotic and, as a solution, used co-axial needles to print fibers with an inner core of skeletal muscle cells and an outer shell of endothelial cells [26]. A different strategy was recently published by the Lewis group, who instead of a sacrificial support matrix produced embryoid bodies, cerebral organoids and cardiac organoids in large-scale microwell culture and compacted these to a ‘support bath’ consisting of half a billion cells in few milliliters. Via direct dispensing, vascular networks were printed into this functional tissue structure (SWIFT method) [27]. The advantage of this strategy is that cells are already assembled in organoids with established cell-to-cell and cell-to-matrix interactions, why the above-discussed death-inducing Anoikis effect by detachment prior to printing is prevented. With this method, also large numbers of cells are already organized in spheroid bodies which represent a vital basis for forming native tissue [27].

A number of additional bioprinting techniques have been developed in recent years, among them different stereolithography-based/light-assisted methods for multi-material/bioink printing via multi-material extrusion heads [28] and SLA to pattern different cell types [29]. The use of light absorbers to prevent unwanted polymerization by scattered light allows high-resolution stereolithography of multi-vascular networks as demonstrated by the development of a vascularized alveolar model [4]. These different methods are beyond the scope of this mini-review and discussed in more detail in recent review articles [1,30,31].

## Post-processing of bio-engineered tissue equivalents

The term ‘4D printing’ is commonly used for 3D-printed material that changes its shape or properties upon external stimuli or over time [32]. From this definition, bioprinting per se is a 4D printing process as, in the cultivation period following biofabrication, the embedded cells adhere to the hydrogel-matrix, proliferate and migrate, cluster, and reorganize themselves to build cellular structures. These processes are steered by the concerted action of the different cell types, ECM-proteins, cytokines, media and growth factors in the bioink, and by external stimuli such as mechanical forces, cultivation at the air–liquid interface or electrical stimulation. Therefore, for each specific tissue equivalent the post-processing procedures will have to be specifically adapted and tissue-specific bioreactors to appropriately stimulate the tissue equivalent are necessary.

## 3D bioprinted human tissue for drug screening, drug validation and optimization and the replacement of animal experiments in medical research

**Figure 3:**
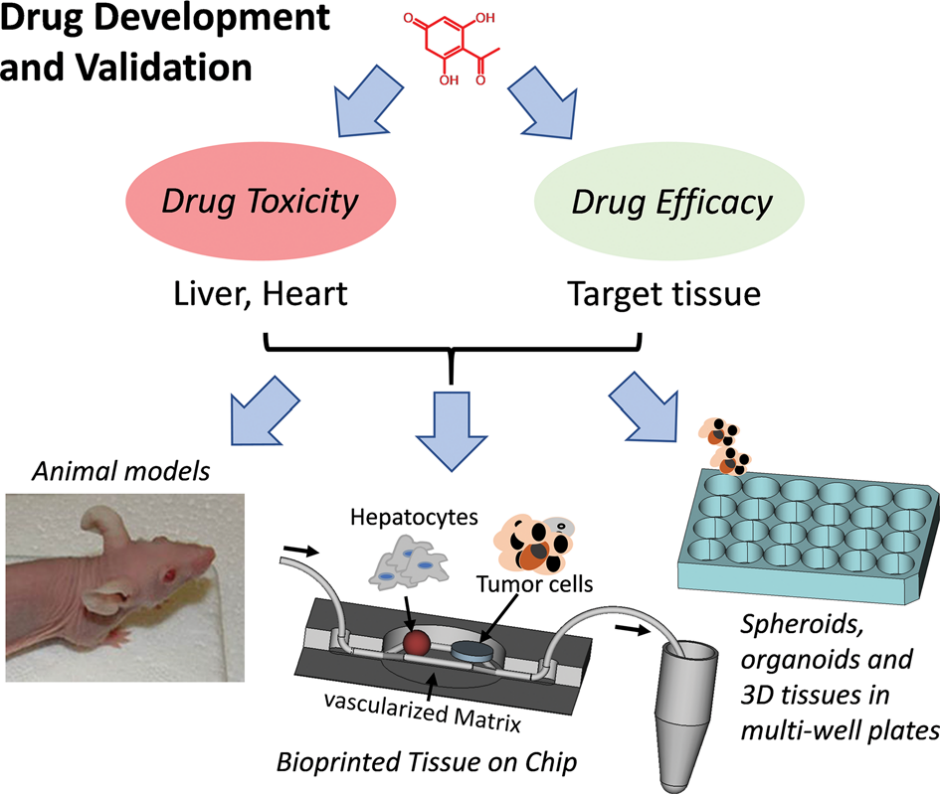
Drug development and drug validation strategies to reduce animal experiments Drug efficacy and drug toxicity profiles can be determined on medium-throughput level via tissue-specific spheroids, organoids or 3D bioprinted mini-tissues in multi-well plates. Read-outs for drug efficacy and toxicity are drug-target specific luminescence-based reporter assays (such as split-luciferase to measure protein–protein interaction in cells), viability assays and microscopy. Alternatively, tissue equivalents can be directly assembled in fluidic chip devices to achieve distinct tissue architectures or combine different tissue types. The overall goal is to refine parameters and reduce or even replace animal experiments by using human tissue surrogates.

**Figure 4:**
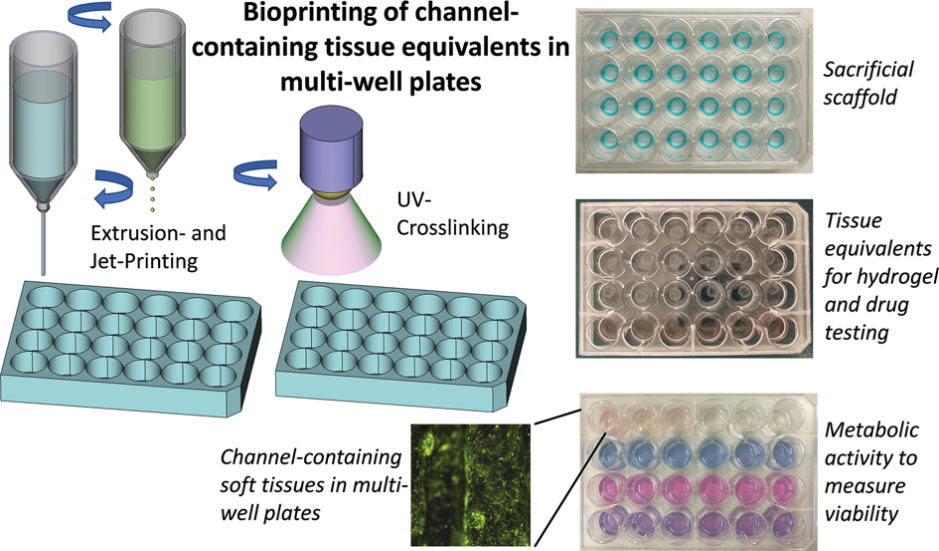
Bioprinting of conduit-containing tissue equivalents in multi-well plates To evaluate biocompatibility of new hydrogel formulations or to assess drug efficacy and drug toxicity we usually bioprint multiple tissue discs in 24-well plates. Standardized cylindric structures are formed by direct-dispensing using the sacrificial substance Pluronic F127. Into these structures, channel-containing, cell laden hydrogel discs are printed using direct-dispensing and micro-jet printing techniques. A main component of these tissues is GelMA that can be polymerized by UV-light-activated photoinitiators, which allows reproducible layer-by-layer assembly of tissue equivalents. Shown is the additive manufacturing of fibroblast-containing tissue-discs in 24-well plates with the (empty) Pluronic F127 scaffolds (top), tissue discs (middle) and metabolic activity measurements at different treatments (bottom). EYFP-expressing human fibroblasts were visualized by fluorescence microscopy.

In drug development, pharmacological efficacy and toxicity profiles of candidate compounds have to be carefully validated to optimize dosage before animal experiments (Figure 3). Most of these *in vitro* studies are still done in 2D cell systems so that many candidate drugs finally fail as cell-physiology in 2D does not reflect the situation in the body. On the other hand, due to non-physiologic test systems, effective drug candidates might be discarded and therefore never enter further development and optimization. However, to mimic tissue response to a specific compound treatment, the complex cellular composition of human tissue including micro-vascularization should be replicated. Organoids, cell spheroids and microfluidic devices represent strategies to better mimic cell-to-cell interaction in 3D; however, most of these systems lack tissue architecture similar to tissues in living organisms. Bioprinting provides this option as tissue can be manufactured in a structured and reproducible way (Figure 4). Although today it is not possible to reproduce human tissue in all of its complexity, bioprinted tissue equivalents have significant advantages over drug testing in animals:

First, bioprinted tissue is directly accessible at every time point during drug treatment thus allowing monitoring of tissue-specific physiologic drug responses, such as reorganization of microvessels or cell death. Changes can be directly imaged by fluorescence microscopy or measured via luminescence-based reporters. This is important to measure drug efficacy on the target tissue, e.g. tumor tissue treated with anti-cancer drugs, but also to assess drug toxicity on for example liver tissue that is exposed to the drug and its by-products [33]. Second, drug metabolites generated in the tissue equivalent or cytokines expressed in response to compound treatment are present in the supernatant or flow-through and can be subjected to further (bio)chemical analyses, which is especially important for drug-exposed liver tissue. Thereby, direct ‘real-time’ drug-conversion, generation of by-products or cytokine expression can be assessed, which is almost impossible in experimental animals. Third, evolution-related physiologic differences between rodents and humans do not influence the outcome of e.g. toxicity studies. This is in particular important in case of liver tissue, as drug responses in mice are not representative for humans due to significant differences in liver cytochrome p450 metabolism [34–36].

Therefore, besides significant attempts to replace animal experiments in the European Union (Directive 2010/63 of the European Parliament) and other countries, the species-specific differences in liver metabolism require complex, human-cell based liver models for drug development, toxin testing and liver fibrosis research [37]. In this respect, it was demonstrated that the patterning of hepatocytes and endothelial cells into lobule-like geometries significantly improves the maturation of hepatic modules [38] and sustains liver function *in vitro* [39]. When transplanting hepato-organoids generated from human HepaRG cells into mice with induced liver failure, it was shown that such organoids are functional and significantly prolong the survival of the animals [40]. Further, bioprinting technology offers fascinating options that cannot be addressed in animals such as to investigate the role of biochemical gradients [41]. The spatio-temporal patterning of cells, hydrogels and growth factors within the tissue equivalent can generate gradients of cytokines that e.g. attract vessels [42], immune cells or regulate wound healing [43]. This potential of bio-assembled tissue was exploited for angiogenesis/osteogenesis studies [44], for high-throughput screening of microenvironment conditions [45] and for the printing of centimeter-scale gastrointestinal tissue [46].

The better understanding of how tumors develop, how they regulate nutrient supply and angiogenesis, their response to cancer therapy and the role of the tumor environment for drug-resistance are highly relevant for cancer treatment and prevention of disease relapse [47,48]. Drug resistance of cancer cells is strongly affected by direct interaction with the surrounding tissue or paracrine stimulation via tumor-associated fibroblasts [49]. In fact tumor cells also reprogram the tumor environment to serve the requirements for further growth and expansion. For decades, cancer cell lines were grown in 2D on plastic surfaces, but it is clear that these models do not replicate the *in vivo* situation in a patient and drugs that may be effective on cancer cell lines fail or elicit adverse side effects as soon as they enter the clinics. Mimicking the complexity of tumor–tumor environment interaction is difficult with traditional tissue engineering techniques, why 3D-patterning of cells and growth factors by bioprinting and the vascularization of the tumor model offers novel options to study the underlying mechanisms. Good examples for this approach are recently published glioblastoma models, where bioinks containing decellularized ECM were combined with glioblastoma cells and endothelial cells to mimic stroma–tumor interactions and patient-specific drug resistance [50] or the recruitment and polarization of macrophages was studied [51]. The combination with e.g. liver tissue [39,40] or bioprinted kidney [52] models will further improve drug efficacy profiles before entering animal models.

Finally, also better conformity and reproducibility in manufacturing fluidic tissue chips compared with conventional methods can be achieved by bioprinting complex tissue equivalents into fluidic chips, until now mostly heart and liver tissue [53–57]. The advantages of ‘tissue chips’ lies in the handling, imaging and analyses of the printed tissue equivalents and in the option to combine different tissues on one platform to e.g. directly assess drug efficacy on target tissue when the drug has passed before a liver surrogate (Figure 3). Especially, this combination of bioprinted tissue equivalents and fluidic chip technology will significantly advance *in vitro* drug discovery and drug validation in future.

## Concluding remarks

3D bioprinting offers fascinating new perspectives for the *in vitro* engineering of living, human tissue for both, organ replacement and drug discovery. It significantly advances current cell culture and spheroid technology via 3D-spatiotemporal patterning of cells and tissue structures. Compared with drug validation in animals, bioprinted tissue equivalents are constructed entirely from human cells, thereby preventing inter-species related biases. This is in particular important for testing of novel anticancer drugs that undergo different chemical modifications in the liver tissue of mice and humans by cytochrome p450. Various bioprinting strategies were developed in recent years. Therefore, we highlight some of the most important recent advances and hope to fascinate the reader for this exciting, vibrant research field.

## Summary

- 3D bioprinting of human tissue is a rapidly evolving field that will change the understanding of cellular organization in complex tissues and reduce or even replace animal experiments in medical research.
- Significant advances in bioprinting methodology in the last few years achieved complex, vascularized tissue equivalents and generated models to mimic liver, heart, skin and many other tissues.
- The technology has the potential to revolutionize tissue repair, patient-tailored therapies and to expedite drug development and validation processes in future.

## Abbreviations

CAD: computer-aided design
ECM: extracellular matrix
FDM: filament deposition modeling
GelMA: gelatin-methacrylate
PCL: polycaprolactone
PU: polyurethane
RGD: arginine-glycine-aspartate-motive
SLA: stereolithogaphy
STL: Surface Tesselation Language/STereoLithography
UV: ultraviolet light
